# Current research status and trends of potassium-competitive acid blockers in the treatment of acid-related diseases: a bibliometric analysis

**DOI:** 10.3389/fphar.2024.1477633

**Published:** 2025-01-07

**Authors:** Baoqiang Zhu, Long Chen, Xue Tao, Hong Zheng, Xia Li, Qingfang Wu, Enwu Long, Haixia Lin

**Affiliations:** ^1^ Department of Pharmacy, The First People’s Hospital of Shuangliu District, West China (Airport) Hospital of Sichuan University, Chengdu, China; ^2^ Department of Pharmacy, Personalized Drug Therapy Key Laboratory of Sichuan Province, Sichuan Academy of Medical Sciences and Sichuan Provincial People’s Hospital, School of Medicine, University of Electronic Science and Technology of China, Chengdu, China

**Keywords:** potassium-competitive acid blocker, acid-related diseases, bibliometric, CiteSpace, VOSviewer

## Abstract

**Objective:**

To explore the current research status and trends of potassium-competitive acid blockers (P-CABs) in the treatment of acid related diseases (ARDs) using bibliometric analysis.

**Materials and methods:**

We collected publications related to P-CAB in the treatment of acid-related diseases in the Web of Science (WOS) Core Collection from the establishment of the database to 30 June 2024. We evaluated the publication volume and citation status over the years using the WOS platform, and visualized the authors, countries, institutions, keywords, and citations of the publications using CiteSpace and VOSviewer.

**Results:**

This study included a total of 455 articles. The number of publications and citations related to research has been increasing year by year. The results show that the scholars with the highest number of publications mainly come from South Korea and Japan. Scholars such as Geun Seog Song, Bongtae Kim, and Nobuhiro Inatomi produced many works in related fields. The most popular drug in this field was vonoprazan, and research on this drug mainly focused on the effectiveness and safety evaluation of ARDs such as *Helicobacter pylori* infection, gastroesophageal reflux disease, peptic ulcers, etc. Researchers were concerned about the evaluation of treatment regimens and efficacy comparison between P-CABs and traditional proton pump inhibitors (PPIs) in the treatment of ARDs. At the same time, researchers are also closely monitoring the potential adverse reactions and long-term adverse outcomes of clinical application of P-CABs for ARDs.

**Conclusion:**

The clinical application of P-CABs, represented by vonoprazan, in ARDs is receiving widespread attention from researchers. The exploration of the application of this type of drug in ARDs is constantly expanding, and it is a research field with great clinical value and research potential.

## Introduction

Acid related diseases (ARDs) are a type of disorder related to gastric acid secretion, involving multiple organs such as the esophagus, stomach, and duodenum. As a common gastrointestinal disease, ARDs mainly include gastroesophageal reflux disease, peptic ulcer, *Helicobacter pylori* infection, and the use of nonsteroidal anti-inflammatory drugs (NSAIDs) (Inatomi et al., 2016; Mori and Suzuki, 2019). Since the launch of proton pump inhibitors (PPIs), they have been widely used in related disease fields as the preferred therapy for ARDs due to their powerful acid suppressing effect. However, PPIs have certain limitations in clinical application. For example, PPIs have delayed efficacy, low bioavailability, and fast metabolism. In addition, this type of drug may be influenced by drug interactions, which may be inconvenient for patients to use (Domingues et al., 2023). The demand and development of acid inhibitors in clinical practice have been ongoing.

Potassium-competitive acid blocker (P-CAB) is a new type of acid inhibitor that can competitively and reversibly block the potassium ion binding site of gastric H, K-ATPase. It can also inhibit the resting and activated H, K-ATPase in cells, and can stay in the secretory tubules at the top of parietal cells for a long time, continuously blocking newly synthesized H, K-ATPase and ultimately producing a long-lasting inhibitory effect on gastric acid secretion (Sugano, 2018; Scott et al., 2015). P-CABs have more significant pharmacological and pharmacokinetic advantages compared to PPIs: fast onset of action without requiring acid activation and specific administration timing, more potent and prolonged inhibition of acid secretion, including a better nighttime acid control, and a less antisecretory variability (Martinucci et al., 2017). It has received widespread attention for its ability to overcome the drawbacks of PPIs. At present, several P-CABs (Blair, 2024; Garnock-Jones, 2015; Kang, 2023) have been approved for market and some P-CABs have been recommended by authoritative guidelines for the treatment of ARDs (Kamada et al., 2021; Zhou et al., 2022). Although P-CABs have shown certain advantages in pharmacodynamics and pharmacokinetics, their effectiveness and safety compared to traditional acid suppression therapy, as well as the feasibility of combining them with other drugs, are still issues worth considering. There is currently no bibliometric research to sort out and analyze relevant studies in order to better grasp the development trends of this type of research.

Bibliometrics is an interdisciplinary science that aims to use mathematical and statistical methods to conduct quantitative and qualitative analysis of various knowledge carriers, in order to enable scholars in related fields to understand the research status and development trends of specific fields (Dai et al., 2023; Ellegaard and Wallin, 2015). Software that can conduct bibliometric research includes CiteSpace (Synnestvedt et al., 2005), VOSviewer, R package “bibliometrix”, etc (Arruda et al., 2022). These software can analyze literature publications during specific time periods and present the relationships between relevant authors, countries, institutions, keywords, and citation situations in the form of graphs (Dai et al., 2023). We are usually accustomed to understanding the current development status of a research field through reviews, and often rely on recently published publications to understand current research hotspots, but reviews have certain limitations; 1) The review is often progressiveness because it summarizes the latest research results, but it cannot show readers the changes and development trends of a certain field for decades; 2) The theme of a review may depend on the author’s subjective consciousness, making it difficult to systematically summarize all publications in a particular research field and draw a comprehensive conclusion; 3) The content of the review is more qualitative and cannot quantitatively analyze like bibliometric research. Currently, there are bibliometric research achievements in medical technology applications (Chen G. et al., 2024), disease treatment (Wang et al., 2024), and drug target discovery (Huang et al., 2024), which can help medical researchers quickly grasp the development trends and emerging frontiers in a certain field. Based on this, we utilized two representative software, CiteSpace and VOSviewer, in this study to explore the clinical application status of P-CABs in ARDs, summarize the efficacy and safety results of P-CABs, and explore the latest research frontiers and hotspots. We hope to provide some reference and inspiration for scholars in the field of research ARDs. Notably, this study employs bibliometric methods for trend analysis. While these methods are useful for visualizing trends in the development and areas of interest within a research field, they are not intended to determine causal relationships, evaluate the quality of evidence, or assess clinical utility.

## Materials and methods

### Data collection and search strategies

We chose the Web of Science (WOS) Core Collection as the search platform for our publications, searching for topics such as “potassium competitive acid blocker”, “Vonoprazan”, “tegoprazan”, etc. The specific search strategy was: (TS=(potassium competitive acid blocker) OR TS=(vonoprazan) OR TS=(tegoprazan) OR TS=(revaprazan) OR TS=(keverprazan) OR TS=(Soraprazan) OR TS=(Fexuprazan) OR TS=(Linaprazan) OR TS=(Zastaprazan)). The search scope for literature is from the establishment of the database until 30 June 2024.

### Inclusion and exclusion criteria

We established relevant standards for the inclusion and exclusion of searched literature. Inclusion criteria: English publications related to the treatment of ARDs with P-CABs, including articles and review articles. Exclusion criteria: 1) Literature unrelated to the above topic (The research scope or theme of these publications is not related to the treatment of ARDs with P-CABs); 2) Conference abstracts, letters, and other types of literature; 3) The language of the publication is not English.

### Literature screening and data collection

Two researchers independently conducted literature screening and decided that a third party would make a decision at any time. For the included literature, we set the record content as “full record and cited references”, exported them in plain text file format for CiteSpace analysis, and exported them in tab delimited file format for VOSviewer analysis.

### Data conversion, cleaning and analysis methods

We used the citation analysis function provided by the WOS platform to obtain publication volume and citation data. By exporting the data, we analyzed the publication and citation trends of the included literature based on Microsoft Excel. For tab delimited file, they can be directly imported into VOSviewer 1.6.19 software. For plain text files, we rename all downloaded documents to “download_**.txt” and saved them in the “input” folder. We used CiteSpace 6.3.R1 software to convert the data for subsequent analysis.

Using Citespace 6.3.R1, the converted data was analyzed for author, institution, country, keyword co-occurrence, keyword clustering, keyword burst and citations. We calculated frequency and centrality of nodes in various indicators through software, where nodes represent authors, countries, institutions, keywords, and references in different indicator. The main parameter settings were as follows: set the time slice to 1 year, the node type to single node, the k value in g-index was 25. When conducting keyword occurrence and reference analysis, “Pathfinder” and “Pruning sliced networks” were selected from the “Pruning” option to prune the network graph. In addition, to ensure meaningful results, we merged keywords with the same meaning, mainly by defining the keyword with the highest frequency as the primary keyword and the other keywords with the same meaning as secondary keywords, and adding the secondary keywords to the primary keywords. The list of merged keywords can be found in Supplementary Table S1.

In VOSviewer 1.6.19, we constructed co-occurrence graphs with author, country and institution frequency thresholds of 5. We selected the counting method as full record and also generated graphs for network visualization and density visualization under different indicators.

## Results

### Annual publication volume and citation results

**FIGURE 1:**
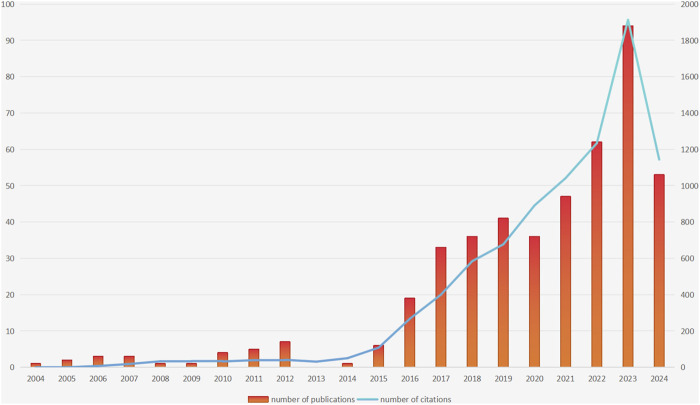
The trend of the number of publications and citations over the years.

This study initially searched for 1086 publications; After setting the included literature types as “article” and “review article”, there were still 730 publications left; Subsequently, 727 articles were left after setting the language type to English. After reading the abstract, 272 articles unrelated to the topic were excluded, and finally 455 articles were included. The annual publication volume and citation results are shown in Figure 1. After analysis, it can be concluded that in terms of publication volume, the earliest publication related to the topic was published in 2004, followed by a significant increase in publication volume in 2016 and reaching its peak in 2023 (The number of publications was 94). Although we only counted publications in the first half of 2024, their publication volume in the first half of the year also reached 53. It is speculated that the publication volume in 2024 will further increase compared to the previous year. The total citation count of the included publications was 8,561 times. There was also a similar trend in terms of citations: the number of citations continued to increase, and reached its peak in 2023 (The number of citations was 1913 times). Based on the above conclusions, we can see that the number of publications and citations related to the study of P-CABs in ARDs are constantly increasing, indicating the growing attention and popularity of this research field.

### Results of co-occurrence analysis of authors

**FIGURE 2:**
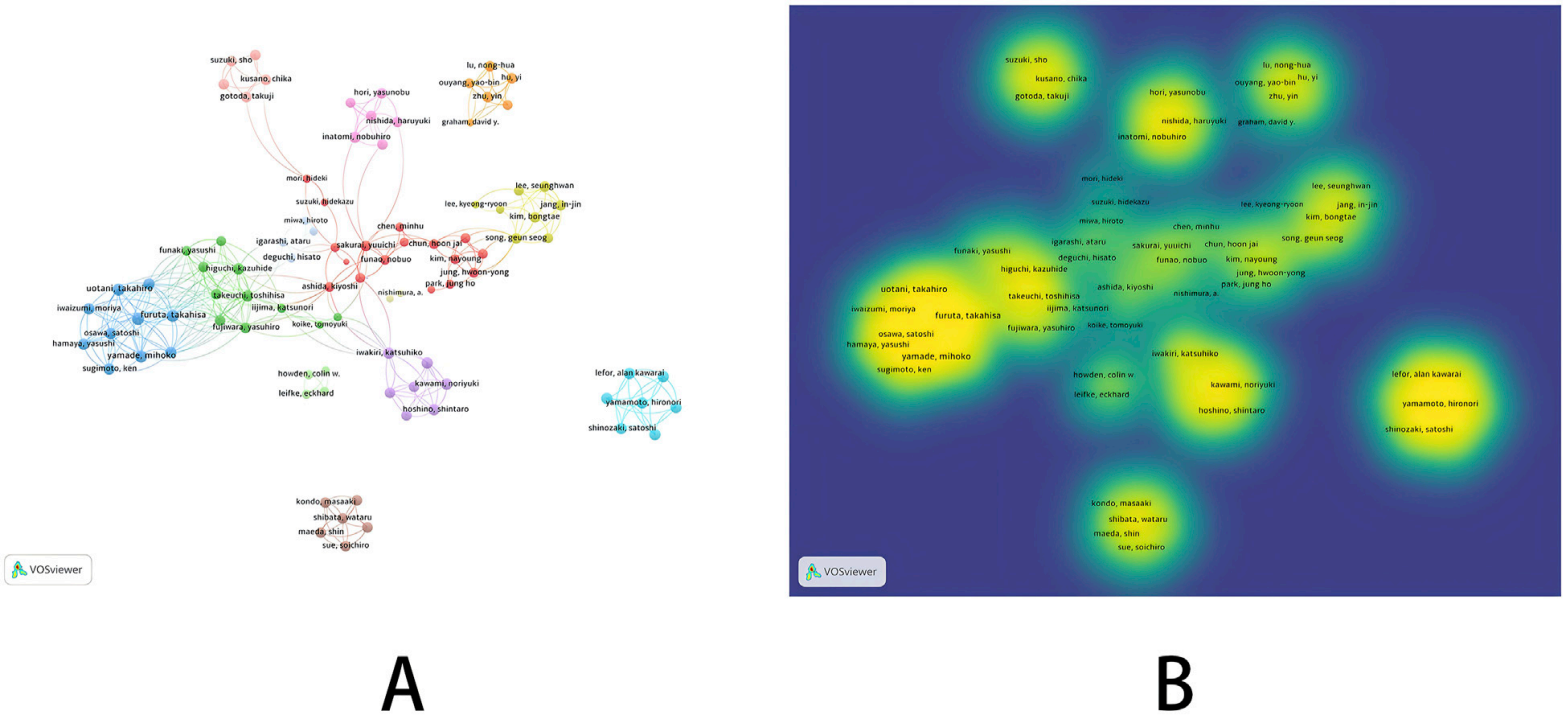
Co-occurrence graph of authors [**(A)** network visualization; **(B)** density visualization].

In the co-occurrence analysis of authors, we used VOSviewer to screen authors with a publication threshold of 5. Among 2,528 authors, a total of 94 authors were selected. The relevant visualization graph is shown in Figure 2, and the list of the top nine authors in terms of publication volume is shown in Table 1. In addition, we constructed a time zone map of author co-occurrence using Citespace, which allows us to directly observe the publication years and collaboration relationships of all authors. Detailed information can be found in Supplementary Figure S1. The top nine authors in terms of publication volume were all from South Korea and Japan, with relatively low centrality. The results of network visualization and overlay visualization (Figures 2A, B) showed that the author collaboration network was generally scattered and formed multiple small groups represented by authors of high publication volume. Considering that the small groups formed by authors with high publication rankings have been engaged in extensive research in related fields, and their publications can to some extent reflect the research trends in this field, we classified and analyzed small groups based on the top nine authors in terms of publication volume.

**TABLE 1:** Top nine authors in terms of publication volume.

No.	Frequency	Centrality	The earliest publication year	Author (country)
1	14	0	2019	Song, Geun Seog (South Korea)
2	13	0	2019	Kim, Bongtae (South Korea)
3	12	0.01	2010	Inatomi, Nobuhiro (Japan)
4	11	0	2017	Iwakiri, Katsuhiko (Japan)
5	11	0	2018	Lee, SeungHwan (South Korea)
6	10	0.01	2018	Jang, In-Jin (South Korea)
7	10	0.03	2015	Sakurai, Yuuichi (Japan)
8	10	0	2016	Shinozaki, Satoshi (Japan)
9	10	0	2016	Lefor, Alan Kawarai (Japan)

Firstly, the collaborative network formed by the research group represented by four researchers from South Korea (Geun Seog Song, Bongtae Kim, SeungHwan Lee, In-Jin Jang) was relatively close. Among the four authors, Geun Seog Song had the highest publication volume (14 articles), followed by Bongtae Kim (13 articles). The time span of the four scholars’ publications is from 2018 to 2023, mainly focusing on the pharmacokinetics, pharmacodynamics, and safety of P-CABs, with the main research object being tegoprazan. As time goes by, researchers’ research directions are constantly changing, and the relevant results mainly involve the combination of tegoprazan and antibiotics (Ghim et al., 2021; Jeon et al., 2021; Huh et al., 2022), the interaction between tegoprazan and food (Han et al., 2021; Yoon et al., 2021), the interaction between tegoprazan and CYP2C19 (Yang et al., 2023), the interaction with NSAIDs (Moon et al., 2022) and effect of acid suppression at night (Han et al., 2023; Yang et al., 2022). Overall, tegoprazan can overcome various pharmacokinetic barriers of previous PPIs administration and can provide a new option for the treatment of ARDs. It is worth noting that in 2023, some scholars explored the carcinogenic potential of P-CABs for long-term acid suppression, and the results showed that tegoprazan induced gastric enterochromaffin-like cell tumors in SD rats (Kim et al., 2023). At the same time, researchers are also working on the development of new formulations of P-CABs, such as the pharmacokinetic differences between the new orally disintegrating tablet of tegoprazan and the conventional tablet (Lee et al., 2023) (The results showed the equivalence of the new formulation), and the pharmacokinetic and pharmacodynamic differences between the delayed-release formulations of tegoprazan and the immediate-release formulation (The results indicated a breakthrough in gastric acid suppression with the delayed-release formulations) (Park et al., 2023).

In addition, among the other five scholars from Japan, Nobuhiro Inatomi had the highest publication volume and the earliest publication time. In 2010, this scholar studied the acid suppressive effect of vonoprazan (TAK 438) in animal models (Hori, 2010) and conducted a series of preclinical studies between 2011–2012 (Hori et al., 2011; Nishida et al., 2012). In 2016, Nobuhiro Inatomi collaborated with Yuuichi Sakurai to systematically elucidate the pharmacological properties of vonoprazan and its potential applications in ARDs (Inatomi et al., 2016; Otake et al., 2016). Yuuichi Sakurai et al. also evaluated the pharmacokinetic differences of the interaction between vonoprazan and other drugs (NSAIDs (Sakurai et al., 2017) or antibiotics (Sakurai et al., 2016)) between 2016 and 2017 to assess the feasibility of P-CABs as a treatment option for gastrointestinal adverse reactions caused by NSAIDs or *Helicobacter pylori* infection. In 2024, the latest study by this scholar evaluated the efficacy of vonoprazan compared to lansoprazole in maintenance therapy for healed erosive esophagitis patients in Asia. The results showed that vonoprazan was not inferior to lansoprazole in preventing the recurrence of erosive esophagitis (Xiao et al., 2024). The articles published by Katsuhiko Iwakiri are all related to gastroesophageal reflux disease, including erosive reflux disease and non-erosive reflux disease. From 2017 to 2019, highly cited research results showed that vonoprazan had more advantages than lansoprazole in the maintenance treatment of healed erosive esophagitis (Ashida et al., 2018), and vonoprazan still had good efficacy in the initial and maintenance treatment of PPIs resistant-reflux esophagitis (Hoshino et al., 2017; Iwakiri et al., 2017; Tanabe et al., 2019). In addition, researchers also paid attention to the resistance mechanism and clinical symptoms of vonoprazan in the treatment of non-erosive reflux disease in the later stage of the study (Kawami et al., 2018; Hoshikawa et al., 2021). Satoshi Shinozaki and Alan Kawarai Lefor had a relatively close cooperative relationship. In the early stages of the study, The research team represented by two scholars focused on the difference in therapeutic efficacy of vonoprazan compared to PPIs for *Helicobacter pylori* infection (Shinozaki et al., 2016; Shinozaki et al., 2018a) (a highly cited evidence-based study showed that the vonoprazan treatment regimen had a significant advantage over PPIs based regimens in second-line *Helicobacter pylori* eradication therapy (Shinozaki et al., 2021)) and the efficacy of vonoprazan in treating gastroesophageal reflux disease (the results showed that vonoprazan could significantly alleviate patients’ gastrointestinal symptoms (Shinozaki et al., 2017; Shinozaki et al., 2018b)). In the later stages of the study, researchers focused on the effects of vonoprazan on serum gastrin levels (Shinozaki et al., 2022a) and gastric morphology. It is worth noting that the results showed that long-term vonoprazan use increased the prevalence of fundic gland polyps, gastric hyperplastic polyps and cobblestone-like mucosa (Shinozaki et al., 2022b). In addition, they explored the therapeutic effect of vonoprazan on functional dyspepsia without heartburn and preliminarily confirmed the short-term efficacy of vonoprazan, similar to that of acotiamide (Shinozaki et al., 2023).

In summary, authors with high publication volumes are concentrated in South Korea and Japan. Although research is scattered, the feasibility of adding P-CABs to ARDs treatment regimens and efficacy for different ARDs are the main focuses. At the same time, researchers also pay attention to the short-term and long-term effects of gastric acid suppression caused by P-CABs treatment.

### Results of co-occurrence analysis of countries and institutions

**FIGURE 3:**
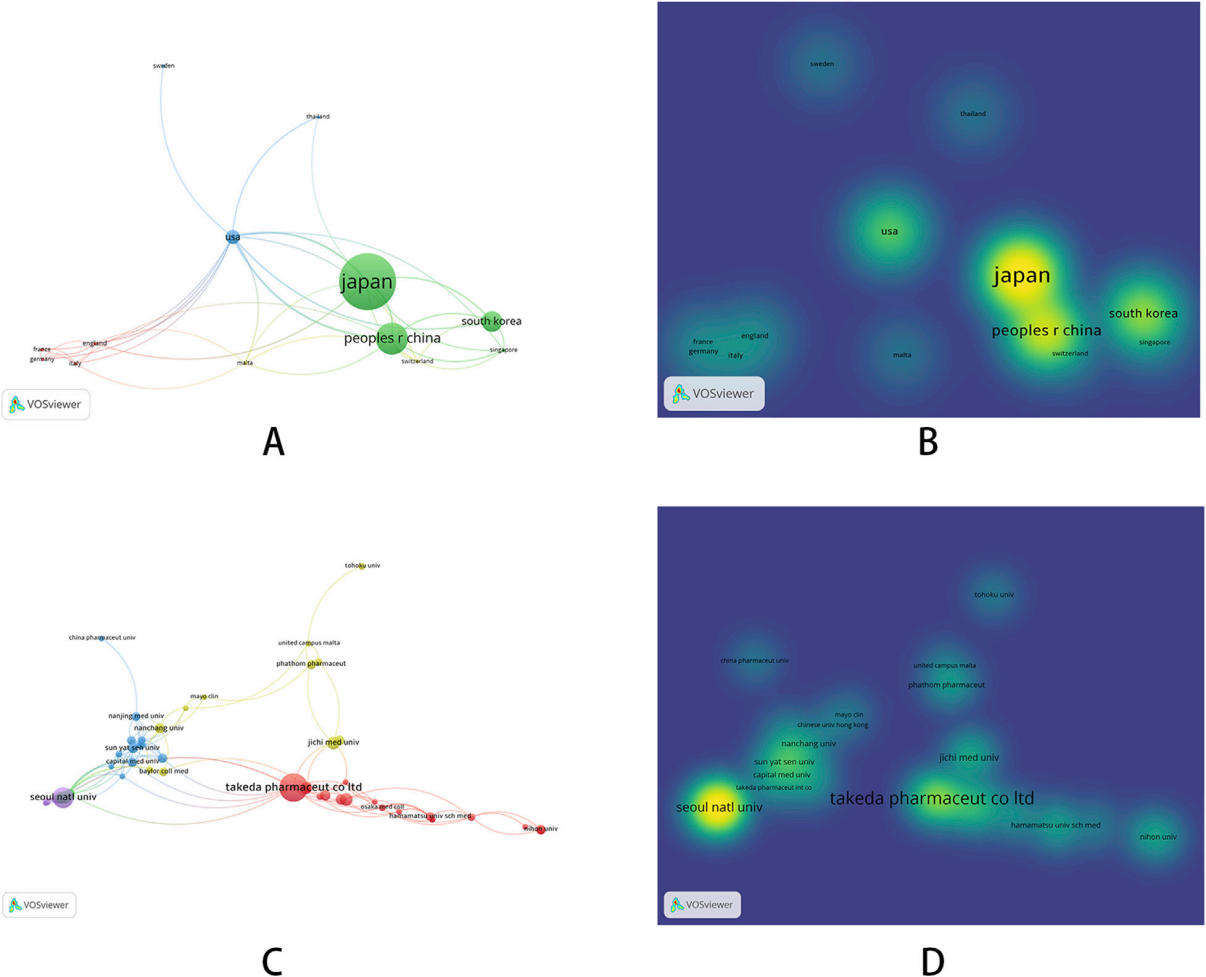
Co-occurrence graph of countries and institutions [**(A)** network visualization of countries; **(B)** density visualization of countries; **(C)** network visualization of institutions; **(D)** density visualization of institutions].

In the co-occurrence analysis of countries, we used VOSviewer to filter countries with a publication threshold of 5. Out of 38 countries, a total of 13 were selected. The relevant visualization graphs are shown in Figures 3A, B, and the top five countries in terms of publication volume are shown in Table 2. We also used CiteSpace to generate a time zone map of country co-occurrence, as detailed in Supplementary Figure S2. Among all countries, the top three in frequency are Japan (210 articles), China (117 articles), and South Korea (77 articles), while the top three in centrality are the United States (0.93), Japan (0.17), and Italy (0.14), indicating that Japan had more output and there was stronger cooperation between the United States and other countries. As the country with the highest number of publications, Japan has reached 12 articles in the first half of 2024. The latest research results focued on the effectiveness of P-CABs in preventing gastrointestinal bleeding, but were more specific. For example, they compared the incidence of rebleeding after gastrointestinal bleeding prevention between P-CABs and PPIs, and compared the effectiveness of P-CABs or PPIs in preventing gastrointestinal bleeding after percutaneous coronary intervention in patients with ischemic heart disease under real-world conditions (Abe et al., 2024; Sasaki et al., 2024). Meanwhile, Japanese researchers have also begun to pay attention to the adverse events of P-CABs and have explored them in clinical studies. They studied the correlation between long-term use of P-CABs and cancer (Arai et al., 2024), as well as treatment strategies for microbiota dysbiosis caused by P-CABs (for example, 1-kestose, a prebiotic, prevented the microbiome changes caused by vonoprazan (Furune et al., 2024)). In 2024, scholars from South Korea and Japan conducted quadruple therapy treatments for *Helicobacter pylori* infection, including one P-CAB, two antibiotics, and one bismuth agent (Tungtrongchitr et al., 2024; Cho, 2024). Although the subjects studied are different (tegoprazan in South Korea and vonoprazan in Japan), it can be inferred that scholars are still searching for the best combination therapy for treating *Helicobacter pylori* infection.

**TABLE 2:** Top five countries in terms of publication volume.

No.	Frequency	Centrality	The earliest publication year	Country
1	210	0.17	2010	Japan
2	117	0.11	2011	Peoples R China
3	77	0.01	2010	South Korea
4	52	0.93	2005	United States
5	10	0.02	2006	England

In the co-occurrence analysis of institutions, we used VOSviewer to filter institutions with a threshold of five for publication volume. Out of 752 institutions, a total of 69 institutions were selected. The relevant visualization graphs are shown in Figures 3C, D. The top ten institutions in terms of publication volume are all from South Korea and Japan, as shown in Table 3. We also used CiteSpace to generate a national co-occurrence time zone map, as detailed in Supplementary Figure S3. Among all institutions, the top three in terms of frequency are Takeda Pharmaceutical Company Ltd. (49 articles), Seoul National University (28 articles), and Catholic University of Korea (15 articles), while the top three in terms of centrality are Takeda Pharmaceutical Company Ltd. (0.35), Hyogo Medical University (0.14), and Chinese Academy of Medical Sciences Peking Union Medical College (0.13). Takeda Pharmaceutical Company Limited is the manufacturer of vonoprazan fumarate tablets (trade name: Vocnti). In 2010, the company published an article introducing the pharmacological effects and basic research results of vonoprazan. Subsequently, when vonoprazan was launched in 2015, the company’s publication volume increased and reached its peak in 2017. The most cited article of the company was published in 2015, which evaluated the efficacy of vonoprazan as a component of first-line and second-line regimens for eradicating *Helicobacter pylori* and demonstrated its noninferiority. In the past 2 years, scholars from the institution have also focused on the safety of using vonoprazan for the treatment of ARDs after its launch. For example, the safety of using vonoprazan for long-term maintenance treatment of erosive esophagitis (Haruma et al., 2023) and the safety of long-term use of vonoprazan in preventing gastrointestinal ulcer recurrence in patients using NSAIDs (Kawai et al., 2023) have been studied, and the results showed that vonoprazan had good safety. At the same time, researchers conducted pharmacokinetic studies on the use of a quadruple therapy containing vonoprazan and bismuth agents in Chinese patients with *Helicobacter pylori* infection, demonstrating the safety of this therapy (Miao et al., 2023).

**TABLE 3:** Top 10 institutions in terms of publication volume.

No.	Frequency	Centrality	The earliest publication year	Institution (country)
1	49	0.35	2010	Takeda Pharmaceutical Company Ltd. (Japan)
2	28	0	2011	Seoul National University (South Korea)
3	15	0.1	2010	Catholic University of Korea (South Korea)
4	14	0.01	2016	University of Tokyo (Japan)
5	14	0	2016	Jichi Medical University (Japan)
6	13	0.01	2017	Hanyang University (South Korea)
7	13	0	2018	Jeonbuk National University (South Korea)
8	13	0.01	2017	Sungkyunkwan University (South Korea)
9	13	0.01	2016	Nippon Medical School (Japan)
10	12	0.14	2016	Hyogo Medical University (Japan)

### Results of co-occurrence analysis of keywords

**FIGURE 4:**
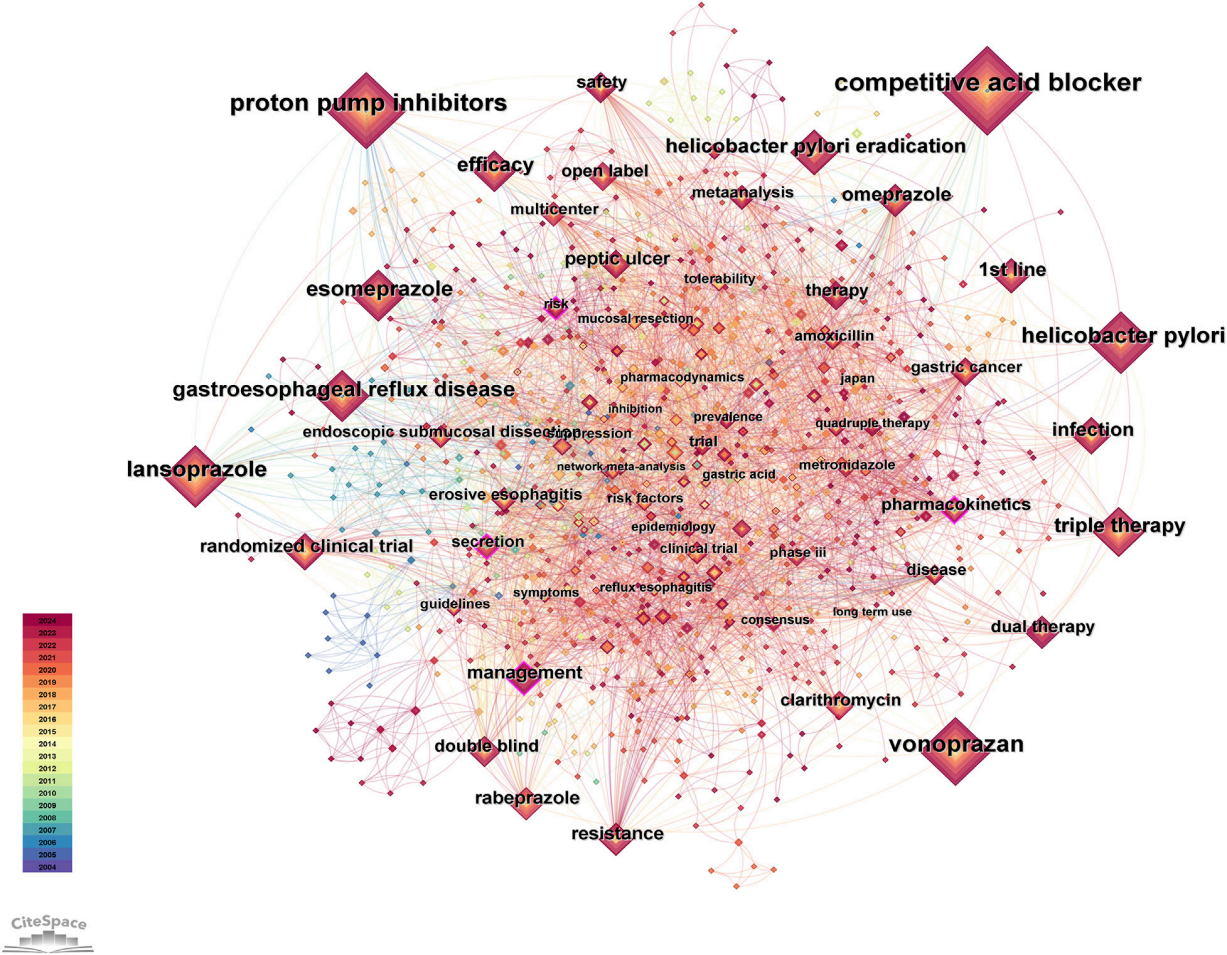
Co-occurrence graph of keywords.

After data cleaning of the keywords, we conducted co-occurrence analysis, cluster analysis, and burst analysis using Citespace. The co-occurrence analysis chart is shown in Figure 4, and the top 30 keywords in terms of frequency are listed in Table 4. In the co-occurrence chart, a total of 981 nodes and 4,917 lines were obtained. Most nodes have low centrality, but the contours of four keyword nodes appear purple (representing centrality greater than 0.1), with centrality of 0.11, namely, “management”, “pharmacokinetics” “secretion” and “risk”. The most frequent keywords can be roughly divided into the following categories: 1) P-CAB, with the most frequent keyword being “vonoprazan” (154). This keyword first appeared in 2014, but its frequency is high, indicating that the drug received high attention during its development and after its launch. 2) ARD, with high-frequency disease keywords including *Helicobacter pylori* infection, gastroesophageal reflux disease, peptic ulcer and erosive esophagitis. In addition, there are multiple related keywords involved in *Helicobacter pylori* infection, including eradication of *Helicobacter pylori*, antibiotic treatment (such as clarithromycin), and resistance issues. 3) PPI, among which high-frequency keywords include “lansoprazole” (135), “esomeprazole” (97), “omeprazole” (61), etc. 4) Drug treatment plans, such as first-line therapy, dual therapy, and triple therapy. 5) Clinical research methods, such as randomized controlled trial, meta-analysis, etc. In addition, “gastric cancer” (33) is also a top 30 keyword, which first appeared in 2016, and research on this topic includes multiple aspects. Several studies evaluated the efficacy of vonoprazan compared to PPIs in the treatment of gastric ulcers after endoscopic submucosal dissection for gastric cancer, and the results showed that vonoprazan has a comparable effect to PPIs (Tsuchiya et al., 2017; Hirai et al., 2018). It is worth noting that a study in 2024 evaluated the differences in the long-term risk of gastric cancer among patients treated with *Helicobacter pylori* eradication regimens containing different acid inhibitors. The results showed that long-term or high-dose use of P-CABs was associated with an increased risk of gastric cancer compared to H2 receptor antagonists (Arai et al., 2024). Based on the content classified by the above keywords, it can be inferred that in the early stages of research, the study of P-CABs focused on drug mechanism, pharmacodynamics and pharmacokinetics. Since the representative drug was put into clinical application, research tended to compare P-CABs with traditional treatment regimens in ARDs, and a large number of clinical trials have been conducted to explore their efficacy and safety. In addition, since the keywords listed in Table 4 all appeared before 2017, we also selected the keywords with the highest frequency ranking and a frequency threshold of 10 (Supplementary Table S2) each year after 2017 for analysis. These keywords appeared later and had a higher frequency, which can help us understand the research trends in recent years. The keyword “risk” (19) has a high centrality, and research related to this topic includes multiple aspects. We have noticed some new findings in highly cited articles, such as the impact of P-CAB use on gut microbiota (there is still controversy over whether vonoprazan affects indomethacin-induced small intestinal injury by altering the gut microbiome) (Nadatani et al., 2019; Li et al., 2022), the safety of P-CAB in children (Kusano et al., 2018), and the gastric changes caused by P-CAB use (Shinozaki et al., 2022b). In addition, studies have found a significant correlation between the use of vonoprazan and *Clostridium difficile* infection (Watanabe et al., 2021). The keyword “network meta-analysis” (11) first appeared in 2019, and in the past 2 years, the number of related publications has increased rapidly, reaching seven articles. Researchers are enthusiastic about using this method to compare the efficacy of multiple drug regimens in treating ARDs (Rokkas et al., 2021; Miwa et al., 2019; Gao et al., 2020). The keyword “quadruple therapy” (14) first appeared in 2020, and in the past 2 years, the number of related research publications has reached 9. The main research focus is on the efficacy of related treatment regimens for *Helicobacter pylori* infection (Lu et al., 2023; Yan et al., 2024).

**TABLE 4:** Top 30 keywords in terms of publication volume.

No.	Frequency	Centrality	The earliest publication year	Keyword
1	267	0.02	2005	competitive acid blocker
2	214	0.01	2004	proton pump inhibitors
3	154	0	2014	vonoprazan
4	148	0	2004	*helicobacter pylori*
5	135	0.04	2006	lansoprazole
6	114	0.03	2005	gastroesophageal reflux disease
7	97	0.01	2011	esomeprazole
8	87	0.01	2015	*helicobacter pylori* eradication
9	79	0.01	2016	triple therapy
10	72	0.01	2008	efficacy
11	61	0.07	2006	omeprazole
12	58	0.01	2016	1st line
13	58	0.02	2016	infection
14	51	0.04	2016	resistance
15	50	0.03	2005	peptic ulcer
16	49	0.04	2015	randomized clinical trial
17	48	0.04	2016	rabeprazole
18	47	0.11	2010	management
19	42	0.06	2016	open label
20	41	0.02	2016	clarithromycin
21	39	0.11	2006	pharmacokinetics
22	36	0.02	2012	double blind
23	35	0.02	2016	dual therapy
24	35	0.05	2015	safety
25	34	0.08	2010	therapy
26	33	0.08	2016	gastric cancer
27	31	0.07	2016	multicenter
28	30	0.07	2016	erosive esophagitis
29	29	0.11	2007	secretion
30	28	0.05	2016	meta-analysis

**FIGURE 5:**
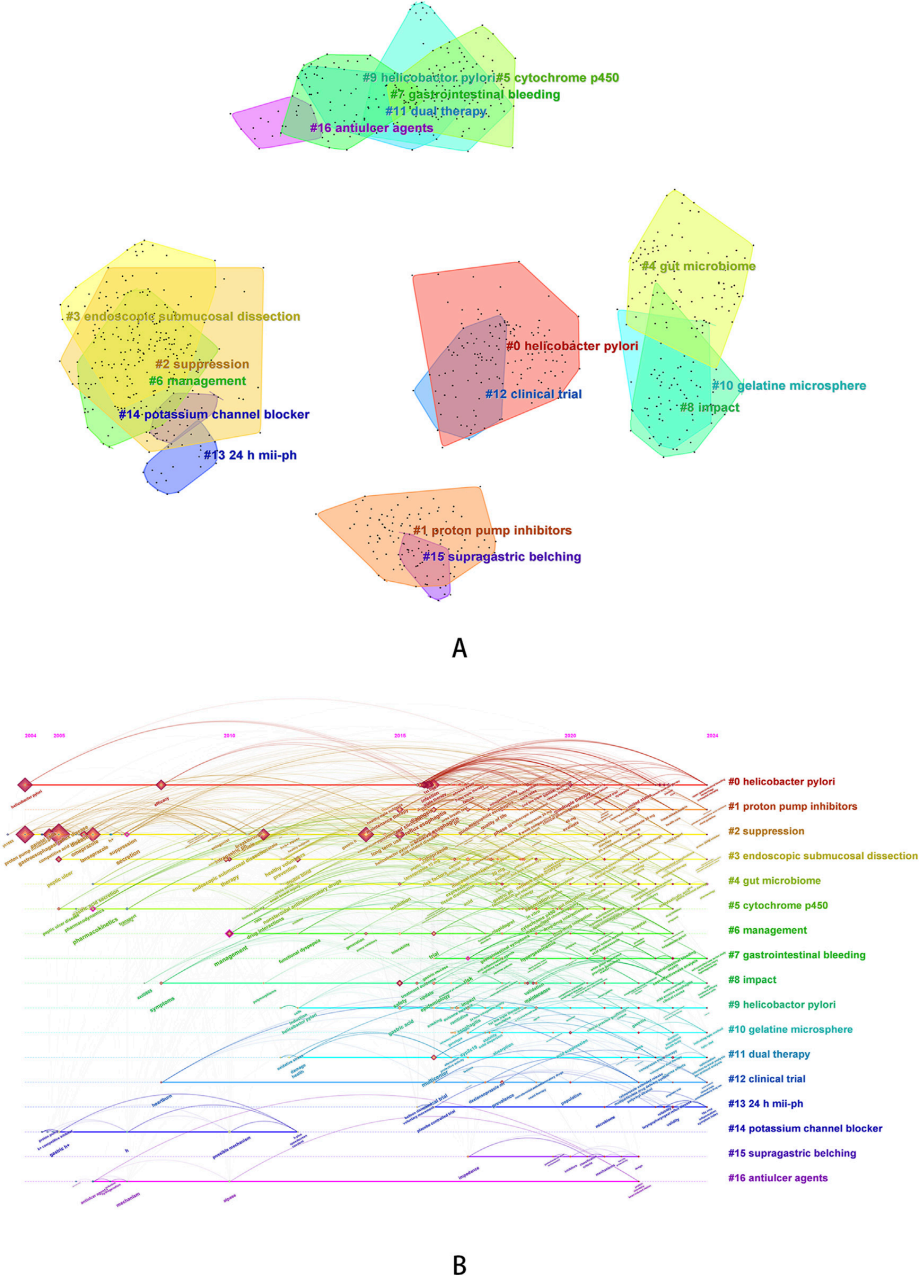
Keyword clustering analysis [**(A)** keyword clustering diagram; **(B)** timeline graph of keyword clustering].

We used the log-likelihood ratio (LLR) method to cluster keywords and further summarize the exploration directions of researchers. A total of 17 clustered words were obtained and ranked according to the number of keywords covered, numbered from # 0 to # 16 in sequence. The clustering diagram of keywords and the timeline diagram of clustered keywords are shown in Figure 5. The line colors in the timeline graph (Figure 5B) correspond to the filling colors of the contours of each cluster word in the clustering graph (Figure 5A). Nodes on each timeline represent the order in which keywords appear over time, and lines represent the relationships between each keyword. The average Modularity Q value is 0.6262 (>0.3), and the average Silhouette S value is 0.8410 (>0.5), indicating that the clustering is reasonable (Sabé et al., 2024).

In the clustering keywords, #1 (proton pump inhibitors, average year: 2020), #10 (gelatine microsphere, average year: 2020), #11 (dual therapy, average year: 2019), #14 (potassium channel blocker, average year: 2007), and #16 (antiulcer agents, average year: 2011) can be classified as therapeutic drugs or treatment regimens. It can be inferred that researchers have been paying attention to anti-ulcer drugs for a long time, and have been focusing on the development of acid inhibitors targeting potassium channels for an earlier period in the past. Around 2015, research including PPIs began to rise and continue to this day, and the comparative results of the two types of drugs continued to receive attention. In addition, researchers have also focused on the development of new formulations, such as gelatin microspheres loaded with revaprazan, which effectively improve the solubility and bioavailability of P-CABs (Kim et al., 2018). In terms of treatment regimens, the topic of dual therapy is relatively late on the timeline, with an average year of 2019. In the past 2 years of research, dual therapy has also received attention in relation to microbial dysbiosis and the use of probiotics (Peng et al., 2023). #0 (*Helicobacter pylori*, average year: 2019), #7 (gastrointestinal bleeding, average year: 2021), #9 (Helicobactor pylori, average year: 2017), and #15 (supragastric belching, average year: 2020) can be classified as diseases or diagnoses. *Helicobacter pylori* infection is a disease that researchers have been continuously concerned about, and the themes surrounding it cannot be separated from infection eradication, drug-resistant treatment and evaluation of treatment plan. PPIs have been used in the past to prevent gastrointestinal bleeding caused by various reasons. In recent years, researchers have also evaluated the feasibility of P-CABs as an intervention in the treatment of cardiovascular and cerebrovascular diseases (Sasaki et al., 2024; Ishii et al., 2023). In addition, as one of the clustering words, supragastric belching is mainly related to vonoprazan-refractory reflux symptoms. Researchers were also concerned about the incidence and mechanisms of behavioral disorders, including supragastric belching and rumination syndrome, in this group of patients (Hoshikawa et al., 2021).

In addition to the above clustering words, We also obtained eight other clustered words. Some of the clustering words, such as # 2 (suppression), # 6 (management), # 8 (impact), and # 12 (clinical trial), represent general themes. We speculated that they are related to acid inhibition of ARDs, treatment of P-CABs, and disease management, so we did not analyze them. We also obtained some clear and representative clustering words, such as #3 (endoscopic submucosal dissection, average year: 2018), #4 (gut microbiome, average year: 2019), #5 (cytochrome p450, average year: 2018) and #13 (24 h mii-ph: 2020). Represented by articles with high citation frequency, researchers evaluated the efficacy of vonoprazan in treating gastric ulcers after endoscopic submucosal dissection and revealed that its efficacy is not inferior to PPIs (Hamada et al., 2019; Maruoka et al., 2017), evaluated the effect of vonoprazan combined with antibiotic therapy (Hu et al., 2022) on the gut microbiota of patients with *Helicobacter pylori* infection and the value of combined probiotic therapy (Kakiuchi et al., 2020), and evaluated the *in vitro* and *in vivo* interactions (Wang et al., 2020) of vonoprazan with other drugs based on cytochrome P450 (such as voriconazole (Shen et al., 2020), proguanil (Funakoshi et al., 2019), etc.). Our clustering analysis also included the clustering term “24-h mii ph”. 24-h multi-channel intraluminal impedance pH, abbreviated as “24-h mii ph”, is a diagnostic criterion for laryngopharyngeal reflux. Previously, this disease is usually treated with PPIs, but in 2024, researchers have begun to explore the effects of P-CABs on the pharyngeal microbiota composition of such patients (Zheng et al., 2024).

Burst analysis can reflect the activity level of certain keywords during a certain period of time, and thus infer the research frontiers and hotspots in different time periods. We chose a γ value of 1.0, a minimum duration of 2 years for keywords, and default values for other parameters to obtain 10 burst keywords (Table 5, where red and blue bars represent keywords bursts and disappearances, respectively). In terms of drug treatment mechanism research, three hot keywords appeared in 2007 and disappeared before 2016. During this period, research focused on exploring the pharmacological effects and activities of P-CABs. Some highly cited studies investigated the affinity and mechanism of action of P-CABs with H, K-ATPase, compared the activity of drugs in different acidic environments (Scott et al., 2015; Luo et al., 2014), and compared the acid suppressive ability of vonoprazan and PPIs in animal models (Hori, 2010; Arikawa et al., 2012). Between 2014 and 2017, “vonoprazan”, “tolerance”, and “pharmacodynamics” became hot keywords, followed by “Japan” from 2016 to 2019. During this period, vonoprazan had been approved and put into clinical use, undoubtedly generating a large number of high-level research results. In terms of pharmacodynamics and tolerability, the most notable studies during this period were the efficacy and tolerability of vonoprazan compared to lansoprazole in gastrointestinal ulcers (Miwa et al., 2017) and erosive esophagitis (Ashida et al., 2015). The results showed that vonoprazan was not inferior to lansoprazole and had good tolerability. In addition, high-quality studies conducted between 2017 and 2018 had shown that vonoprazan had non inferiority compared to lansoprazole in the maintenance treatment of erosive esophagitis (Ashida et al., 2018). For patients with erosive esophagitis who were ineffective with PPIs, vonoprazan still had a certain effect (Iwakiri et al., 2017).

**TABLE 5:** Top 10 keywords with the strongest citation bursts.

Keywords	Year	Strength	Begin	End	2004–2024
Secretion	2007	4.39	2007	2016	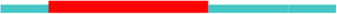
Suppression	2007	4.34	2007	2015	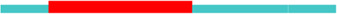
H, K-ATPASE	2007	3.16	2007	2015	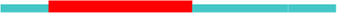
Omeprazole	2006	4.64	2008	2012	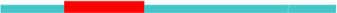
Vonoprazan	2014	4.02	2014	2017	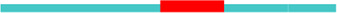
Tolerability	2015	5.29	2015	2017	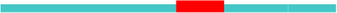
Pharmacodynamics	2006	4.17	2015	2017	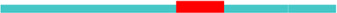
Japan	2016	4.55	2016	2019	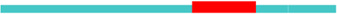
Erosive esophagitis	2016	3.38	2017	2018	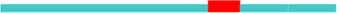
Rabeprazole	2016	3.28	2019	2021	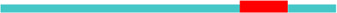

### Results of co-citation analysis

**FIGURE 6:**
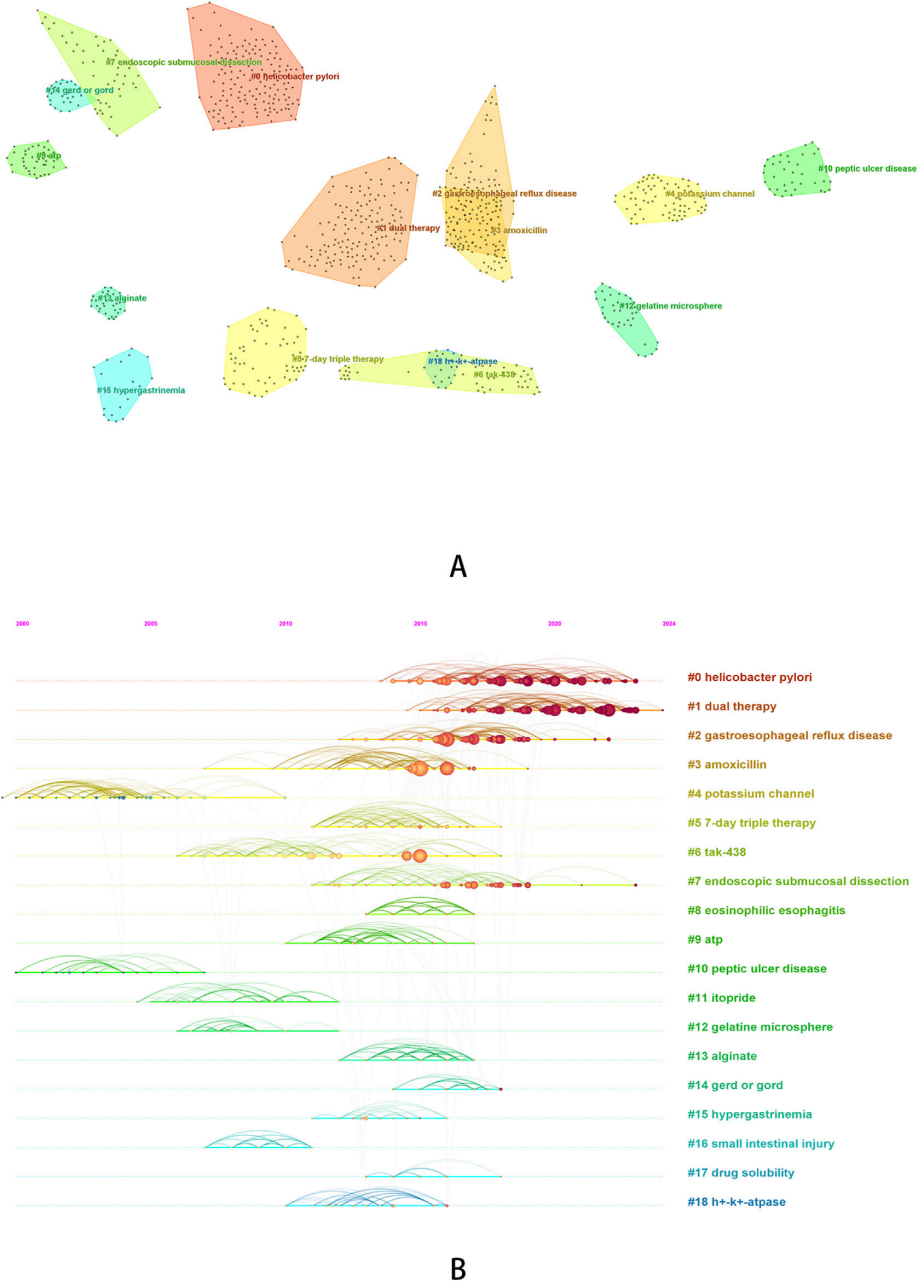
Cluster analysis of co-cited reference [**(A)** cluster diagram of co-cited reference; **(B)** timeline graph of co-cited reference clustering].

Citation analysis is a statistical method of illustrating the quantity of cited studies. Generally, the more often a study is cited, the greater its impact. Co-citation is when two studies are cited by ≥1 study at the same time, which indicates how closely the two studies are related. The greater the number of studies cited simultaneously, the closer the relationship between the two (Jiang et al., 2024). Based on co-citation analysis, we can gain a deeper understanding of the research trends and hotspots that researchers in this field are concerned about. Therefore, we conducted a co-citation analysis (The top five cited references are shown in Table 6), and at the same time, we performed cluster analysis (Figure 6) and burst analysis (Table 7) on the cited literature. The top five cited references were all studies related to vonoprazan, published in 2015–2016. Two studies (Sakurai et al., 2015; Jenkins et al., 2015) conducted in healthy male subjects in 2015 demonstrated that vonoprazan had a rapid onset of action, good tolerability, and a faster and more sustained acid suppressive effect than PPIs. A 2016 study (Murakami et al., 2016) showed that vonoprazan had a higher first-line eradication rate compared to lansoprazole when added to first-line triple therapy for the treatment of *Helicobacter pylori* infection. In addition, a pharmacogenomics study in 2016 showed that the use of vonoprazan 20 mg twice daily was not affected by CYP2C19 genotype and was superior to esomeprazole in drug metabolism (Kagami et al., 2016).

**TABLE 6:** The top five publications with the highest citation count.

No.	Frequency	Publication year	Cited publication
1	81	2015	Acid-inhibitory effects of vonoprazan 20 mg compared with esomeprazole 20 mg or rabeprazole 10 mg in healthy adult male subjects--a randomised open-label cross-over study (Sakurai et al., 2015)
2	81	2016	Vonoprazan, a novel potassium-competitive acid blocker, as a component of first-line and second-line triple therapy for *Helicobacter pylori* eradication: a phase III, randomised, double-blind study (Murakami et al., 2016)
3	74	2015	Randomised clinical trial: safety, tolerability, pharmacokinetics and pharmacodynamics of repeated doses of TAK-438 (vonoprazan), a novel potassium-competitive acid blocker, in healthy male subjects (Jenkins et al., 2015)
4	73	2016	Randomised clinical trial: vonoprazan, a novel potassium-competitive acid blocker, vs. lansoprazole for the healing of erosive oesophagitis (Ashida et al., 2016)
5	60	2016	Potent acid inhibition by vonoprazan in comparison with esomeprazole, with reference to CYP2C19 genotype (Kagami et al., 2016)

**TABLE 7:** Top 19 references with the strongest citation bursts.

References	Year	Strength	Begin	End	2004–2024
Matsukawa J, 2011, BIOCHEM PHARMACOL, V81, P1145, DOI 10.1016/j.bcp.2011.02.009	2011	11.16	2011	2016	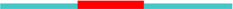
Hori Y, 2011, J PHARMACOL EXP THER, V337, P797, DOI 10.1124/jpet.111.179556	2011	9.39	2011	2016	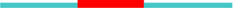
Hori Y, 2010, J PHARMACOL EXP THER, V335, P231, DOI 10.1124/jpet.110.170274	2010	7.82	2011	2015	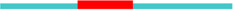
Jenkins H, 2015, ALIMENT PHARM THER, V41, P636, DOI 10.1111/apt.13121	2015	17.84	2015	2020	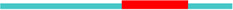
Sakurai Y, 2015, CLIN TRANSL GASTROEN, V6, P0, DOI 10.1038/ctg.2015.18	2015	11.01	2015	2020	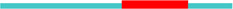
Ashida K, 2015, ALIMENT PHARM THER, V42, P685, DOI 10.1111/apt.13331	2015	10.41	2015	2019	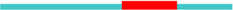
Garnock-Jones KP, 2015, DRUGS, V75, P439, DOI 10.1007/s40265-015-0368-z	2015	4.99	2015	2020	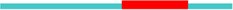
Sakurai Y, 2015, ALIMENT PHARM THER, V42, P719, DOI 10.1111/apt.13325	2015	20.05	2016	2020	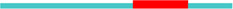
Ashida K, 2016, ALIMENT PHARM THER, V43, P240, DOI 10.1111/apt.13461	2016	11.91	2016	2021	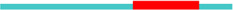
Kagami T, 2016, ALIMENT PHARM THER, V43, P1048, DOI 10.1111/apt.13588	2016	9.75	2016	2021	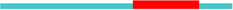
Shinozaki S, 2016, KAOHSIUNG J MED SCI, V32, P255, DOI 10.1016/j.kjms.2016.04.009	2016	4.79	2016	2020	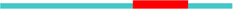
Murakami K, 2016, GUT, V65, P1439, DOI 10.1136/gutjnl-2015-311304	2016	14.41	2017	2021	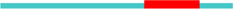
Suzuki S, 2016, AM J GASTROENTEROL, V111, P949, DOI 10.1038/ajg.2016.182	2016	6.57	2017	2021	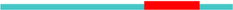
Sakurai K, 2017, WORLD J GASTROENTERO, V23, P668, DOI 10.3748/wjg.v23.i4.668	2017	4.76	2017	2021	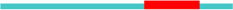
Matsumoto H, 2016, DIGEST DIS SCI, V61, P3215, DOI 10.1007/s10620-016-4305-0	2016	4.76	2017	2021	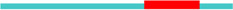
Tsujimae M, 2016, DIGESTION, V94, P240, DOI 10.1159/000454762	2016	4.76	2017	2021	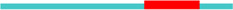
Otake K, 2016, ADV THER, V33, P1140, DOI 10.1007/s12325-016-0345-2	2016	3.63	2017	2021	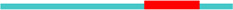
Hoshino S, 2017, DIGESTION, V95, P156, DOI 10.1159/000456072	2017	4.97	2018	2022	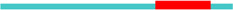
Li M, 2018, *HELICOBACTER*, V23, P0, DOI 10.1111/hel.12495	2018	4.7	2019	2024	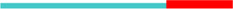

Based on the clustering analysis of cited literature, we obtained a total of 19 clusters, The average Modularity Q value is 0.8834 (>0.3), and the average Silhouette S value is 0.9497 (>0.5), indicating that the clustering is reasonable (Sabé et al., 2024). We can roughly divide the 19 clustered words into several categories. #0 (*helicobacter pylori*), #2 (gastroesophageal reflux disease), #8 (eosinophilic esophagitis), #10 (peptic ulcer disease), #14 (gerd or gord) can be classified as ARDs, and based on the previous analysis, we can also classify research topics related to #7 (endoscopic submucosal dissection) as ARDs. It can be observed that research on *Helicobacter pylori* infection has been widely cited in recent years, and compared to other diseases, research on *Helicobacter pylori* infection remains a hot topic. #1 (dual therapy), #3 (amoxicillin), #5 (7-day triple therapy), #6 (tak-438), #11 (itopride), #12 (gelatine microsphere) and #17 (drug solubility) can be classified as therapeutic drugs or treatment regimens. Among them, research on dual therapy has been widely cited in recent years and has received higher attention compared to triple therapy. #4 (potassium channel), #9 (ATP), and #18 (h + - k + - ATPase) can be classified as research mechanisms, and they are earlier in the timeline. In addition, we also found some other clustering words: #13 (alginate), #15 (hypergastrinemia), #16 (small intestinal injury). The publications under these clustered words have been jointly cited over a period of time, and the studies that cited these publications specifically focused on the adverse reactions of hypergastrinemia caused by P-CABs (Sugano, 2018) and the potential protective effects of P-CABs on small intestine injury (Lim et al., 2012).

In the burst analysis, most of the studies were published between 2015 and 2016 on vonoprazan, which has already been introduced before, so we will not elaborate on the results. We would like to focus on reporting the last study in the table, which was published in 2018 and has been receiving attention since 2019. This article was a systematic review that mainly evaluated the efficacy of vonoprazan compared to PPIs, added to triple therapy, in treating patients with clarithromycin resistant *Helicobacter pylori* infection. The results demonstrated that vonoprazan was equally effective as traditional PPIs (Li et al., 2018).

## Discussion

Our bibliometric research results indicate that in the past 20 years, scholars have been dedicated to the study of P-CABs in the treatment of ARDs, aiming to overcome the limitations of traditional PPIs therapy in clinical applications and find drugs with stronger acid suppression effects. The successive launch of drugs such as vonoprazan and tegoprazan has attracted the attention of researchers (especially scholars from Japan and South Korea) to the clinical efficacy of P-CABs compared to PPIs. The clinical research scope of P-CABs for the treatment of ARDs is constantly expanding and the research level is deepening. Currently, the clinical research scope is no longer limited to *Helicobacter pylori* infection, gastroesophageal reflux disease, and peptic ulcers caused by NSAIDs. Researchers seem to have explored various acid related diseases that were previously recommended for PPIs treatment (including artificial ulcers, stress ulcers, laryngopharyngeal reflux, functional dyspepsia, etc.). By summarizing the evidence we previously mentioned in the results section, we found that P-CABs are superior to PPIs in terms of acid suppression ability, which is expected to further expand the clinical indications of P-CABs. At present, the research results of P-CABs in ARDs have also promoted the update of clinical guidelines. Taking Japan as an example, in 2022, Japan proposed in the evidence-based clinical practice guidelines for gastroesophageal reflux disease (Iwakiri et al., 2022) that both PPIs and P-CABs can be recommended as first-line treatments for patients with mild reflux esophagitis. In 2020, the Japanese Society of Gastroenterology proposed in its evidence-based clinical practice guidelines for peptic ulcers (Kamada et al., 2021) that PPIs or P-CABs are recommended as first-line drugs for initial non curative treatment of peptic ulcers. In addition, Japan also proposed in the revised management guidelines for *Helicobacter pylori* infection in 2016 (Kato et al., 2019) to use PPIs or P-CABs combined with amoxicillin and clarithromycin or metronidazole triple therapy for 7 days as a treatment plan for *Helicobacter pylori* eradication. These guidelines are based on positive clinical evidence related to P-CABs, which demonstrates that P-CABs may be superior or equivalent to medication regimens involving traditional PPIs in the past.

In recent years, researchers have continued to pay attention to the efficacy of drug combination therapy involving P-CABs in the treatment of *Helicobacter pylori* infection (especially in comparing the efficacy of dual therapy and quadruple therapy, and adjusting antibiotic resistance regimens). In the past 2 years, a large number of studies have compared the efficacy differences of dual therapy containing P-CAB (vonoprazan + amoxicillin), quadruple therapy containing P-CAB and bismuth agents, and standard quadruple therapy containing PPIs in eradicating *Helicobacter pylori*. The results showed that dual (Yan et al., 2024; Wang et al., 2023) or quadruple (Lu et al., 2023; Zhuang et al., 2024) therapy containing P-CAB had advantages equal to or better than traditional quadruple therapy in a treatment course of 10–14 days (Chen C. et al., 2024; Li et al., 2024), and exhibited good tolerability (Huh et al., 2022). These two treatment options have shown some advantages over traditional quadruple therapy, for example, the dual therapy treatment option can provide new choices for areas with clarithromycin resistance or populations with high incidence of adverse reactions, while having lower treatment costs; The quadruple therapy seems to achieve sufficient therapeutic effect at a daily administration frequency of 20 mg (Zhuang et al., 2024) or in a 10 days treatment course (Lu et al., 2023). The course of treatment and frequency of administration seem to be a direction worth considering, especially in areas with different antibiotic resistance rates. More research is still needed to further validate the relevant results.

In addition, scholars are also very concerned about the adverse events caused by P-CABs, such as hypergastrinemia, *C. difficile* infection, gastric mucosal lesions, and imbalance of gut microbiota, as well as the adverse outcomes of long-term use. In previous studies, it has also been found that PPIs cause some similar adverse reactions. Previous studies have shown that PPIs can increase the infection rate of *C. difficile* (Tariq et al., 2017), which may be related to changes in the pH or microbiome of the gastrointestinal tract (Deshpande et al., 2012; Trifan et al., 2017; Imhann et al., 2016). At present, more than one study has simultaneously identified the risk of *C. difficile* infection caused by P-CABs or PPIs, but it is still uncertain whether P-CABs are more susceptible to infection than PPIs due to their stronger acid suppressive effect (Ouyang et al., 2024; Saruta et al., 2023). Meanwhile, long-term PPIs treatment has also been shown to cause hypergastrinemia and increase the incidence of gastric enterochromaffin-like cell hyperplasia (Lundell et al., 2015). Long term use of PPIs is also associated with an increased risk of gastric cancer, with one possible explanation attributed to elevated levels of gastrin and alterations in the microbiome (Arai et al., 2024; Hayakawa et al., 2016). Although basic research on tegoprazan (Kim et al., 2023) and clinical studies on vonoprazan (Arai et al., 2024) have presented such evidence, the latest research has not yet found that P-CABs have a higher risk of developing gastric cancer compared to PPIs. Given the insights from previous research, clinical practice should pay more attention to related risk issues with caution.

This study has certain strengths: 1) Compared to traditional review articles, our research can summarize the included studies through quantitative analysis and objectively present longitudinal research topics that change over time, which can serve as a supplement to review articles and provide readers with more comprehensive information. 2) At present, the drug development and clinical research of P-CABs have been continuously carried out. In our analysis, the research around vonoprazan is the most extensive and comprehensive. Previous research topics and directions may provide valuable insights for the clinical study of P-CABs that have not yet been fully researched.

Meanwhile, our research has certain limitations: 1) Due to software requirements, we can only select one database for analysis. The WOS core collection contains a large number of representative literature, so we chose this database for analysis. However, some literature from other databases cannot be included. 2) Foreign literature is only in English, which may overlook high-quality literature in other languages. In the future, we hope include more comprehensive databases for further exploration. 3) This study identified the overall development trends and primary focuses within the P-CAB research field based on bibliometric analysis. However, bibliometric methods focus on quantitative evaluation, such as citation counts and publication frequency, and cannot elucidate causal relationships, evaluate the quality of individual study designs, or directly demonstrate clinical utility. Therefore, the results of this study should be regarded as supplementary data for providing an overview of research trends. For determining treatment guidelines, more rigorous evidence synthesis, such as meta-analysis, is indispensable.

## Conclusion

In summary, based on bibliometric research, we have reviewed and explored the research trends, development status, and frontiers of P-CABs in the treatment of ARDs. It can be concluded that the clinical application of P-CABs, represented by vonoprazan, in ARDs has attracted widespread attention from researchers, and the exploration of the clinical application of this type of drug is gradually expanding. Positive clinical evidence is also constantly emerging. This is a research field with clinical value and research potential, and in the future, P-CABs may bring a new hope for the treatment of various ARDs.

## Data Availability

The original contributions presented in the study are included in the article/Supplementary Material, further inquiries can be directed to the corresponding authors.

## Abbreviations

ARD, acid-related disease; CYP, cytochrome P450; LLR, log-likelihood ratio; NSAID, nonsteroidal anti-inflammatory drug; P-CAB, potassium-competitive acid blocker; WOS, Web of Science.
