# Supplementary material for: Current research status and trends of potassium-competitive acid blockers in the treatment of acid-related diseases: a bibliometric analysis
Source: Front Pharmacol. 2025 Jan 7;15:1477633. doi: 10.3389/fphar.2024.1477633 (PMC11747516; doi:10.3389/fphar.2024.1477633)
Supplement: Supplementary file 1 [file Table1.docx]

Table S1 List of keyword merging

| **Primary keywords** | **Secondary keywords** |
| --- | --- |
| competitive acid blocker | potassium-competitive acid blocker  potassium-competitive acid blockers  blocker  potassium-competitive acid blocker (p-cab)  potassium competitive acid blocker  potassium competitive acid blockers  potassium channel acid blockers  potasium-competitive acid blocker |
| proton pump inhibitors | proton pump inhibitor  proton-pump inhibitor  proton-pump inhibitors  acid pump antagonist  proton pump inhibitors  pump inhibitor therapy  proton pump inhibitors (ppis)  acid pump antagonists  proton pump inhibitor (ppi) |
| lansoprazole | vs. lansoprazole |
| vonoprazan | tak 438  monofumarate tak 438  tak 438 vonoprazan  vonoprazan fumarate  vonoprazan (vpz)  vonoprazan (tak-438f)  fumarate  clinical trial vonoprazan |
| helicobacter pylori eradication | eradication  eradication therapy |
| gastroesophageal reflux disease | gastroesophageal reflux  reflux disease  gastroesophageal reflux disease (gerd)  gerd  reflux  gastroesophageal reflux symptoms  eflux symptoms |
| helicobacter pylori | helicobacter pylori infection |
| resistance | clarithromycin resistance  antibiotic resistance  primary antibiotic resistance  antimicrobial resistance  amoxicillin resistance  microbial drug resistance  fluoroquinolone resistance |
| disease | diseases |
| risk factors | risk factor |
| rabeprazole | rabeprazole 10 |
| esomeprazole | esomeprazole 40 mg |
| 1st line | 1st line treatment |
| consensus | consensus report |
| gastric cancer | early gastric cancer |
| peptic ulcer | gastric ulcers  gastric ulcer  ulcers  ulcer  duodenal ulcer  duodenal ulcers |


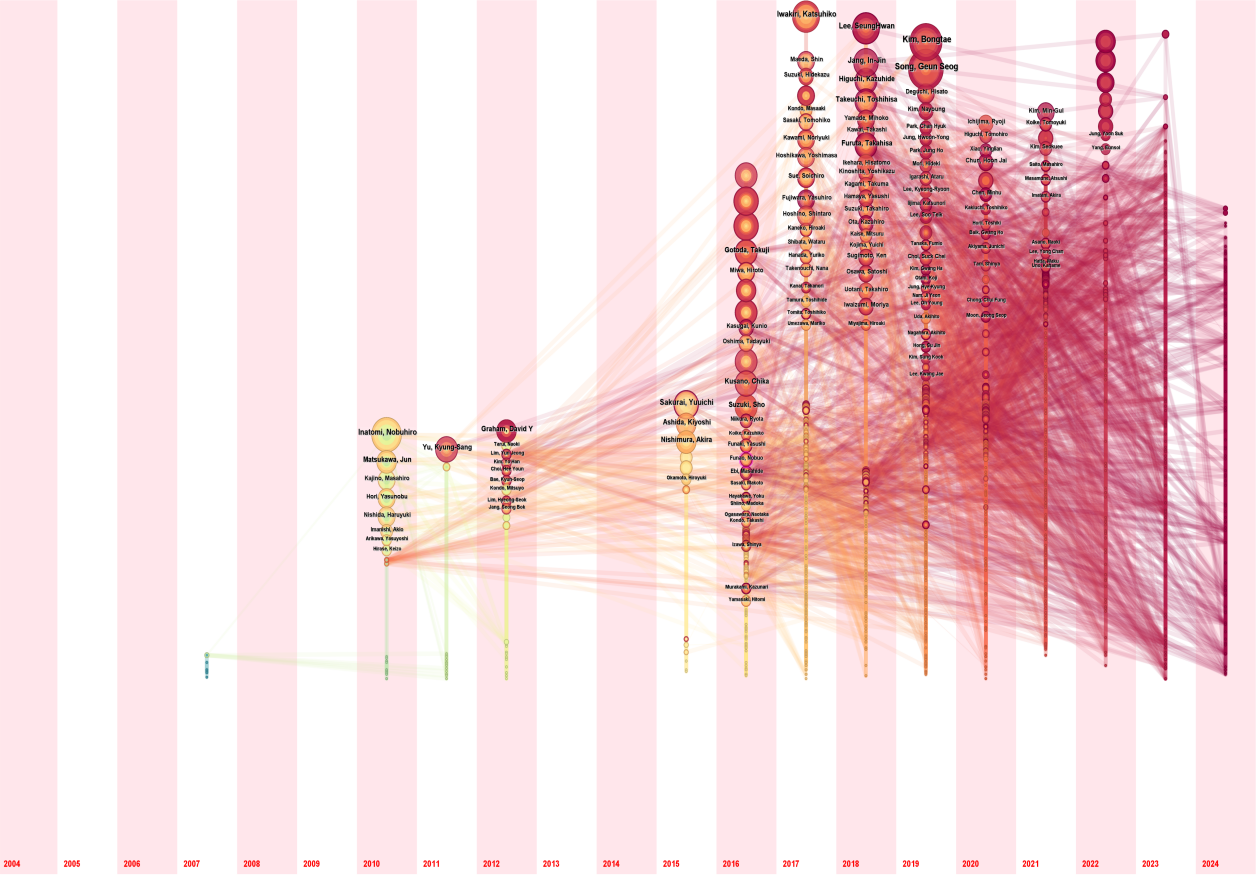
Figure S1 Time zone map for co-occurrence research of authors


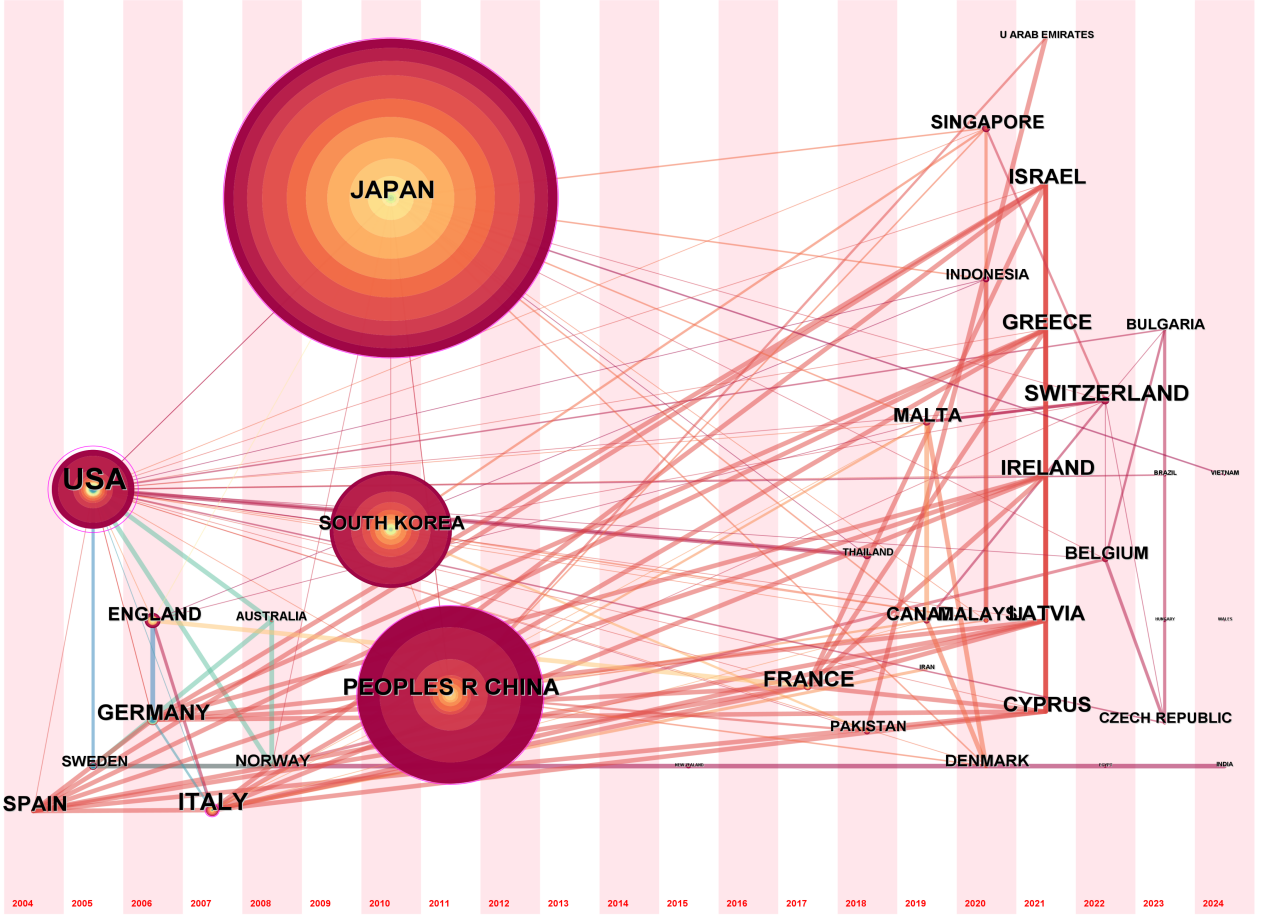
Figure S2 Time zone map for co-occurrence research of countries


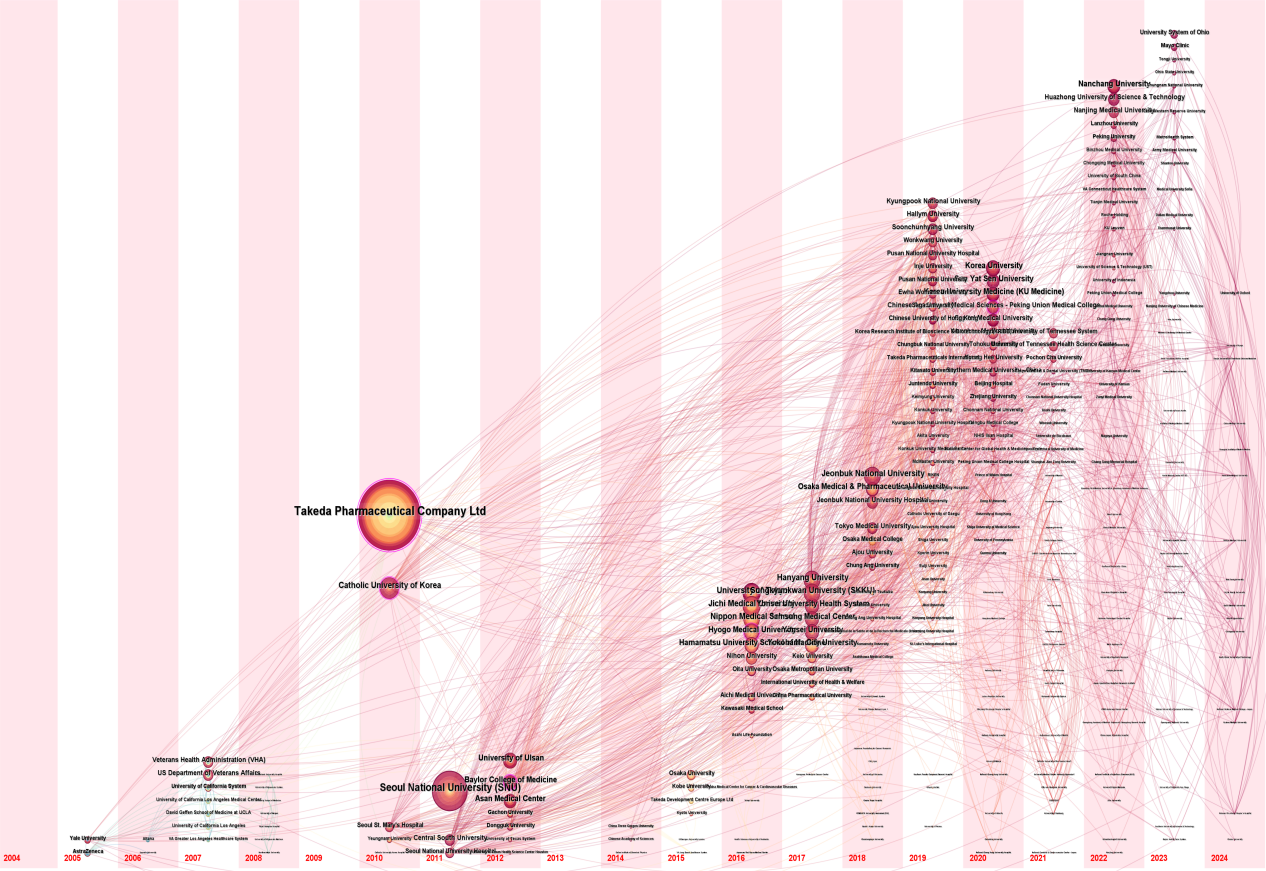
Figure S3 Time zone map for co-occurrence research of institutions

Table S2 Keywords with a frequency threshold of 10 per year after 2017

| **No.** | **Frequency** | **Centrality** | **The earliest publication year** | **Keyword** |
| --- | --- | --- | --- | --- |
| 1 | 19 | 0.11 | 2017 | risk |
| 2 | 17 | 0.05 | 2018 | phase iii |
| 3 | 16 | 0.03 | 2017 | guidelines |
| 4 | 15 | 0.01 | 2017 | mucosal resection |
| 5 | 15 | 0.05 | 2018 | prevalence |
| 6 | 14 | 0.02 | 2020 | quadruple therapy |
| 7 | 11 | 0.03 | 2019 | network meta-analysis |
